# Tapered Cladding Design for Monolithic Waveguide–Photodetector Coupling in Si-Based Integrated Photonics

**DOI:** 10.3390/nano15221731

**Published:** 2025-11-17

**Authors:** Alfredo A. Gonzalez-Fernandez, Jorge A. Vazquez-Hernandez, Felix Aguilar-Valdez, Neil Moffat

**Affiliations:** 1 National Institute for Astrophysics, Optics, and Electronics (INAOE), Puebla P.O. Box 51, Mexico; jorge.vazquez@inaoep.mx (J.A.V.-H.); faguilar@inaoep.mx (F.A.-V.); 2 Institut de Microelectrònica de Barcelona (IMB-CNM, CSIC), Universitat Autònoma de Barcelona, 08193 Bellaterra, Spain; neil.moffat@imb-cnm.csic.es

**Keywords:** integrated photonics, silicon photonics, electrophotonics, nitride photonics, photonic sensors

## Abstract

Silicon photonics offers a powerful route to leverage existing microelectronics infrastructure to enhance performance and enable new applications in data processing and sensing. Among the available material platforms, silicon nitride (Si_3_N_4_) provides significant advantages due to its wide optical transmission window. A key challenge, however, remains the monolithic integration of passive nitride-based photonic components with active electronic devices directly on silicon wafers. In this work, we propose and demonstrate a tapered bottom-cladding design that enables efficient coupling of visible light from Si_3_N_4_/SiO_2_ core–cladding waveguides into planar p–n junction photodiodes fabricated on the silicon surface. Si_3_N_4_/SiO_2_ waveguides were fabricated using fully CMOS-compatible processes and materials. Controlled reactive ion etching (RIE) of SiO_2_ allowed the formation of vertically tapered claddings, and finite-difference time-domain (FDTD) simulations were carried out to analyze coupling efficiency across wavelengths from 509 nm to 740 nm. Simulations showed transmission efficiencies above 90% for taper angles below 30°, with near-total coupling at 10°. Experimental fabrication achieved angles as low as 8°. Responsivity simulations yielded values up to 311 mA W^−1^ for photodiodes without internal gain. These results demonstrate the feasibility of fabricating monolithic Si-based waveguide–photodetector systems using simple, CMOS-compatible methods, opening a scalable path for integrated photonic–electronic devices operating in the visible range.

## 1. Introduction

Integrated photonics promises unprecedented advances in fields ranging from quantum computing to biomedical applications [1,2,3,4,5]. Leveraging well-established silicon (Si) microelectronics technologies not only increases yield and reduces costs but also enables seamless integration of photonic and electronic functionalities. Over the past decade, significant efforts have been devoted to overcoming the intrinsic limitations of Si for photonic applications, such as its indirect band gap and limited transparency range [5,6,7,8].

Notable advances have been achieved through the use of nanostructures for light emission and the incorporation of compatible materials, such as silicon nitride, which presents additional advantages like the widening of the operational wavelength range (*λ*), spanning from visible to mid-infrared [5,9,10]. However, several challenges remain, and improvements in many areas are necessary to achieve truly practical applications. One of the main difficulties in realizing fully monolithic emitter–waveguide–detector systems is the efficient coupling of light between active and passive photonic components. On one hand, strong optical confinement is necessary for low-loss wave guiding, while on the other, efficient transmission into embedded photodetectors is essential for reliable optical-to-electrical signal conversion on the wafer level.

Several approaches have been explored to couple light from waveguides to photodetectors in silicon photonics. The most widely reported include heterogeneous integration of photodetectors [1,5,10,11], edge coupling of Si photodetectors to nitride waveguides on SOI platforms [12], evanescent coupling into undercut regions of Si wafers [8], and modifying the bottom cladding of waveguides to maintain co-planarity with surface photodetectors [4,13,14]. The first three approaches severely limit compatibility with standard Si-based fabrication, as they often require non-CMOS-compatible materials, complex processes, or photodetectors located on planes different from the electronics. The last approach can, in principle, be realized with CMOS processes but introduces significant challenges for light guiding, coupling, and detecting, such as the reported requirement of epitaxial Si layers [13], the need for surface planarization techniques such as chemical–mechanical polishing (CMP) [15], or other energy-intensive reflow methods [14].

In this work, we study a seldomly explored alternative design that enables efficient transmission of light from Si_3_N_4_/SiO_2_ core–cladding waveguides to photodetectors located directly on the surface of Si wafers. The approach relies on controlled tapered etching to facilitate mode coupling and is fully compatible with standard CMOS materials and fabrication techniques. Unlike previous studies, our design specifically addresses the transmission and coupling of visible light, such as that emitted by Si-compatible nanostructure-based light sources [16]. This opens the pathway toward the full monolithic integration of emitter–waveguide–photodetector systems, without the need for Local Oxidation of Silicon (LOCOS) or additional planarization steps for applications that can take advantage of the visible spectrum, such as sensing and biosensing.

We employ finite-difference time-domain (FDTD) simulations to evaluate the structural parameters required to improve waveguide-to-detector transmission. The analysis considers variations in geometry, taper angles, surface roughness, and material thicknesses, all within the process windows of standard CMOS fabrication. Prototype structures were fabricated and experimentally characterized to validate the feasibility of the proposed design, and additional simulations were performed to confirm device operation with the realized geometries. Finally, the responsivity of the proposed waveguide–photodetector structures is compared with previously reported approaches, demonstrating comparable or superior performance while requiring significantly simpler and more cost-effective fabrication processes.

## 2. Materials and Methods

Pilot samples of SiO_2_, with and without Si_3_N_4_ films, were fabricated on standard Si substrates in the clean room facilities of the Institut de Microelectrónica de Barcelona–Centro Nacional de Microelectrónica. A 1.5 μm SiO_2_ layer was either thermally grown or deposited by Plasma-Enhanced Chemical Vapor Deposition (PECVD) on n-type <100> Si wafers. Waveguide patterns with widths ranging from 1 μm to 100 μm were defined using UV lithography and subsequently transferred into the oxide layer by Reactive Ion Etching (RIE). Etching conditions were tuned to obtain different profiles with varying lateral-to-vertical etch ratios, enabling the fabrication of tapered slopes across different samples. A 300 nm-thick Si_3_N_4_ film was then deposited by Low-Pressure Chemical Vapor Deposition (LPCVD).

Step profiles were measured using a DektakXT profilometer. Cross-sectional inspection of the multilayered structures was performed using Focused Ion Beam (FIB) milling combined with Scanning Electron Microscopy (SEM). Surface roughness was analyzed by Atomic Force Microscopy (AFM).

All tests and simulations were conducted and declared at room temperature (23 °C) and local atmospheric pressure (900 hPa).

Optical performance was analyzed using the Finite-Difference Time-Domain (FDTD) method implemented using ANSYS Lumerical FDTD (version 2023 R1) [17]. The excitation source was configured as the TE fundamental mode, calculated beforehand with the MODE solver of the same software package. Transmission and insertion losses were simulated over wavelengths ranging from 509 nm to 740 nm. The analysis considered both the total optical power coupled into the waveguide and the power collected by monitors placed at the wafer surface in the photodiode region.

Responsivity of the full system was modeled using Lumerical CHARGE. The optical generation rate, obtained from the FDTD simulations, was used as the input to calculate the photocurrent generated in a p–n junction, following the device configuration reported in [14]. A reverse bias of 25 V was applied during the photodiode simulations.

## 3. Results

**Figure 1:**
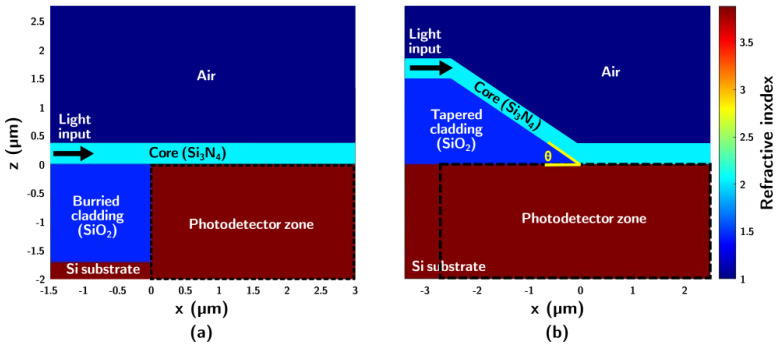
Cross-section of the waveguide-photodetector structures studied. (**a**) Buried bottom cladding approach [14]. (**b**) Ramp-coupling approach.

**Figure 2:**
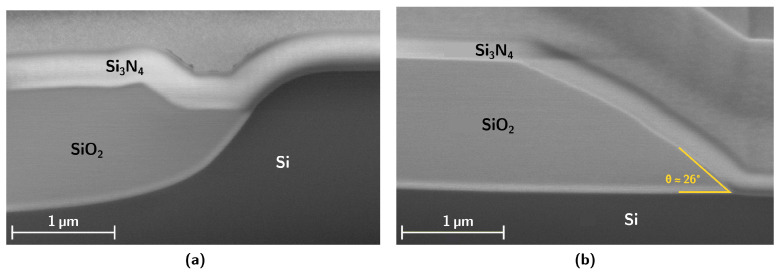
SEM images of samples with buried bottom SiO_2_ cladding as reported in [14] (**a**), and the proposed tapered bottom cladding (**b**).

Figure 1 compares two architectures: the buried cladding approach previously reported in Figure 1a [14,18], and the novel tapered bottom cladding structure proposed in this work Figure 1b. The illustration highlights the idealized features of each design, omitting fabrication-related imperfections. For example, in the buried cladding structure Figure 1a, irregularities such as the bird’s beak effect inherent to LOCOS are disregarded [14], while in the tapered design Figure 1b, the transition from the straight section to the ramp is shown as perfectly sharp. The real features after fabrication can be observed in Figure 2, which provides SEM images of samples obtained by the two approaches. In the tapered cladding approach Figure 2b, the transition is smoother than in the schematic ideal representation, and the ramp profile is not always perfectly linear. On the other hand, Figure 2a shows the buried cladding structure obtained through LOCOS and further planarization using successive borosilicate deposition and etching [14], where a very pronounced irregularity can be observed towards the end of the bottom cladding, which is expected to introduce significant optical disturbance.

The smoother transition from the straight to the ramp region is expected to be beneficial to light transmission, as this reduces leakage to air.

**Figure 3:**
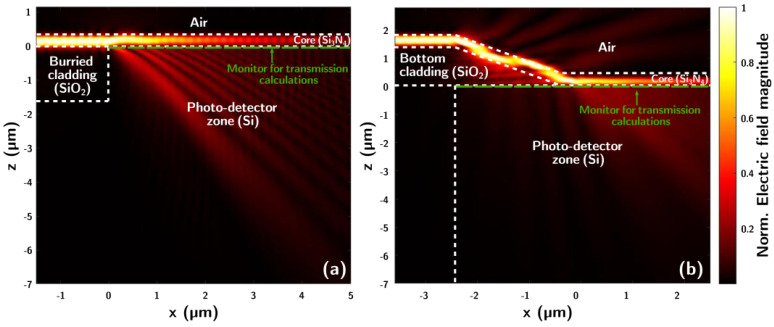
FDTD simulations of light with a wavelength of 633 nm for the buried-cladding approach (**a**), and the tapered-cladding approach (**b**).

Different etching conditions were tested, achieving ramp angles ranging from 8.4° to 26.8°. In order to explore the influence of the ramp angle in coupling, simulations using different values were performed. Figure 3 shows the cross-section field distribution for light with *λ* = 633 nm of a system using the buried cladding approach Figure 3a and for a ramp approach with 26° inclination Figure 3b, which corresponds to the sample presented in Figure 2.

**Figure 4:**
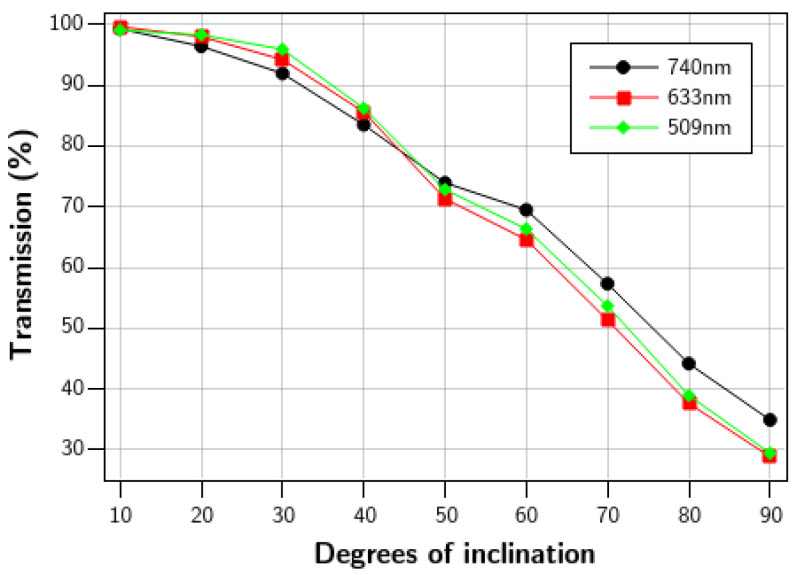
Proportion of the light inserted to the WG which is transmitted to the wafer surface in the region of the photodetector for different waveguide taper angles.

Figure 4 shows the transmission results for simulations at a range of taper angles. Starting from a perfect step of 90° to a taper angle of 10° for three reference wavelengths.

In order to obtain metrics that allow for comparison to similar approaches, such as those reported in [13,19], as well as to different strategies such as the in-line photodetector deposition approach reported in [12], we conducted simulations of the photoelectric response for the coupled light considering the simple p-n junctions reported in [14]. Responsivity values of the structure with a ramp of 8° for *λ* = 633 nm are very similar to those obtained for the buried approach: 0.302 A W^−1^ for the former and 0.311 A W^−1^ for the latter.

## 4. Discussion

Figure 3 shows clear light confinement in the waveguide core up until the Si substrate is reached. For the buried oxide architecture, the absorption of light by the detector starts at the end of the bottom cladding (x = 0 µm in Figure 3a). On the other hand, for the tapered cladding approach, transmission to the substrate starts at some point after the beginning of the ramp descent, clearly before the nitride core makes direct contact with the substrate. The initial point of absorption depends on the ramp angle, which determines the effective thickness of the bottom cladding for every given point. Therefore, to maximize light detection, the photodiode should encompass all the active area. In the case of the buried dioxide system, the photodetector location is limited by the bottom cladding position and can only be placed after this, extended to the point where the field in the core vanishes if the detection is to be maximized. However, the ramp approach allows flexible photodetector placement, since the entire wafer surface is available. As mentioned, the position in which the light is first absorbed will vary with geometry but also with wavelength. This approach is particularly promising for sensing applications. Multiple wavelengths (*λ*) can be simultaneously coupled to a single waveguide, with the taper engineered to selectively filter specific wavelengths and direct them to photodetectors positioned at corresponding locations. In this way, variations in the intensity of a particular *λ* of interest can be monitored, enabling fully integrated spectroscopic sensing methods.

Angle control presents an interesting challenge, since standard CMOS processes are typically optimized to produce sharp steps rather than gradual ramps. Therefore, process conditions must be carefully adjusted to achieve specific angles and, in turn, the desired system behavior. Our fabrication results showed it is possible to fabricate angles as low as 8° by modifying standard RIE parameters, and Figure 4 shows that when the photodiode covers the full ramp length, the wedge design can achieve transmission above 90% for angles lower than 30°, and nearly complete transmission by 10°. The transmission reduction as the angle becomes steeper can be mainly attributed to leakage to the air, which is mostly introduced by the discontinuity of the structure at the beginning of the ramp (knee).

Responsivity simulations for the integrated p–n junction photodiodes indicate competitive performance compared with approaches using other types of photodetectors, including conventional p–n junctions, avalanche photodiodes (APDs), and single-photon avalanche diodes (SPADs). A key advantage of the proposed design lies in the significantly simpler fabrication processes required. In addition, it should be noted that these results correspond to idealized structures. In practice, the buried cladding approach is likely to show reduced performance, mainly due to the bird’s beak effect and thinning of the cladding after planarization, whereas the tapered-ramp approach is expected to improve performance owing to smoother mode transitions between straight and ramped sections.

When comparing the simulation results to experimental reports on other waveguide-photodetector systems reported in the literature, the structures presented here show similar or better response. Similar inverse taper and top-down approaches reported in [13,19] present responsivity values of 0.109 A W^−1^ and 0.304 A W^−1^ for *λ* = 850 nm, and the latter uses a Ge on Si amplifier diode. Regarding reports considering visible light, ref. [12] reports responsivity of 0.830 A W^−1^ for *λ* = 685 nm, almost tripling our best result, but remaining in the same order of magnitude, and its fabrication requires the use of non-standard CMOS Si wafers, epitaxial deposition of Si, chemo-mechanical polishing, and deep-UV lithography, which significantly reduces its suitability for high-yield integrated photonics and reduces its fabless compatibility as compared to the other options.

## 5. Conclusions

The results confirm that nitride-core/oxide-cladding waveguides with ramp-shaped coupling structures can efficiently direct light into integrated photodetectors located on the surface of standard Si wafers fabricated through conventional CMOS technology and materials. This approach is viable across a wide spectral range, from the blue to the near-infrared, and is fully compatible with previously reported Si-based light emitters, showing its potential for complete Si-compatible emitter-waveguide-photodetector systems, making it a promising platform for optical biosensing applications. In particular, the ability to engineer tapered waveguides to filter and guide specific wavelengths to dedicated photodetectors opens the door to compact, integrated spectroscopic sensing.

A key advantage of the proposed method, compared with other integrated waveguide–photodetector systems reported in the literature, lies in its simplicity and cost-effectiveness. It eliminates the need for non-mainstream CMOS processes such as LOCOS, planarization, epitaxy, or CMP, while still achieving comparable responsivity—even without relying on photodetectors with amplification mechanisms.

Overall, this CMOS-compatible strategy offers a scalable and practical route toward low-cost, monolithic photonic–electronic integration, paving the way for future applications in optical communications, quantum technologies, and biosensing.

## Data Availability

The original data presented in the study are available upon reasonable request.
